# miR-146a, miR-146b, and miR-155 increase expression of IL-6 and IL-8 and support HSP10 in an *In vitro* sepsis model

**DOI:** 10.1371/journal.pone.0179850

**Published:** 2017-06-29

**Authors:** Dagmar Pfeiffer, Eva Roßmanith, Ingrid Lang, Dieter Falkenhagen

**Affiliations:** 1 Institute of Cell Biology, Histology and Embryology, Medical University Graz, Graz, Austria; 2 Center of Biomedical Technology, Danube University Krems, Krems, Austria; University of Texas MD Anderson Cancer Center, UNITED STATES

## Abstract

microRNAs (miRNAs) play an essential role in inflammation processes including sepsis. This study aimed to identify miRNAs as candidates for therapies that are involved in the innate immune response and to assess their potential functions in the activation of the endothelium. We stimulated THP-1 monocytes with 10 ng/ml LPS for 4 h and used the supernatant for the stimulation of human umbilical vein endothelial cells (HUVEC) or human pulmonary microvascular endothelial cells (HPMEC) for 16 h. miRNA array analysis (of 1,891 miRNAs) identified a 1.5-fold upregulation of miR-146a, miR-146b, and miR-155 in stimulated endothelial cells. HUVEC were transfected with miRNA inhibitors for miR-146a, miR-146b, and miR-155 to investigate the function of these miRNAs in endothelial inflammatory pathways. Inhibition of miR-146a resulted in a diminished release of interleukin (IL)-6 and IL-8 by respective 68% and 55% (*P*<0.001). Inhibition of miR-146b reduced the expression of IL-6 by 49% (*P*<0.001). Inhibition of miR-155 reduced the expression of IL-6 and IL-8 by respective 31% (*P*<0.001) and 14%. The inhibition of miR-146a, miR-146b, and miR-155 reduced the release of HSP10 by 50%, 35%, and 69% (*P*<0.05), respectively, but did not influence the expression of HSP27 or TXA2. In conclusion, miR-146a, miR-146b, and miR-155 are exerting anti-inflammatory properties by down-regulating IL-6 and IL-8, and influencing the expression of HSP10 in the activated endothelium. We provide evidence for the central role of selected miRNAs in sepsis and their use in the development of small interfering RNA therapeutics to target immune cells and sepsis pathways.

## Introduction

Sepsis is defined as life-threatening organ dysfunction caused by a dysregulated host response to infection [1]. The activated endothelium promotes the expression of pro-inflammatory cytokines, such as IL-6 and IL-8, and initiates the expression of cell adhesion molecules, thus contributing to leukocyte recruitment from the blood into extravascular tissues [2]. In addition, heat shock proteins, such as HSP10, are released from damaged or stressed cells acting as danger signals [3]. Intracellular expression and extracellular release of HSP increase and cause an overzealous activation of immunostimulatory effects in sepsis [4]. HSP10 acts as an antagonist to Toll-like receptor 4 (TLR-4) and exerts anti-inflammatory activity by inhibiting TLR-4-mediated recognition of lipopolysaccharides (LPS) [5].

In the last decade, miRNAs have emerged as potent regulators of vascular inflammation. miRNAs are small, noncoding, double-stranded RNA molecules that are involved in the regulation of gene expression by decreasing mRNA stability or inhibiting mRNA translation [6]. miRNAs act as key regulators in a wide variety of biological processes, such as development, differentiation, and homeostasis [6,7]. In addition, miRNAs are in focus as regulatory elements of the immune system and immune responses [8,9].

The induction of miRNAs by TNF-α, IL-1, and TLR during activation of lymphocytes and monocytes has already been intensively investigated [9]. Nevertheless, the role of miRNAs in the activation of endothelial cells and their influence in the expression of pro-inflammatory cytokines and action of heat shock proteins remains rather elusive.

Endothelial-specific miRNAs influence the expression of endothelial cell adhesion factors [10]. Expression of vascular cell adhesion molecule 1 (VCAM-1) is inhibited by the action of miR-126, whereas miR-31 and miR-17-3p are both known to negatively regulate E-selectin and intercellular adhesion molecule 1 (ICAM-1) [10–12].

We hypothesized that selected miRNAs in stimulated endothelial cells influence the cytokine expression and are involved in the action of heat shock proteins. We believed that selected miRNAs are interesting candidates for new RNA interference-mediated therapies in sepsis by regulating key inflammatory cytokine expression.

## Materials and methods

### Primary cells and cell lines

Primary HUVEC were isolated from umbilical cords according to the method described by Gimbrone [13]. Additionally, cells were selected with anti-CD31 antibodies using magnetic beads (Dynabeads, Dynal Biotech, Oslo, Norway) and cultured in Medium 199 (Sigma-Aldrich, St. Louis, MO, USA) buffered with 20 mM 4-(2-hydroxyethyl)-1-piperazineethanesulfonic acid (HEPES, Sigma-Aldrich) containing 20% fetal bovine serum (FBS; PAA, Pasching, Austria), 10 μg/ml endothelial cell growth supplement (ECGS; BD Biosciences, Franklin Lakes, NJ, USA), 15 IU/ml heparin (Baxter, Vienna, Austria), and 100 μM penicillin-streptomycin (P/S; Sigma-Aldrich). Ethical approval was granted by the ethics committee of the Medical University of Graz (reference EK: 28–333 ex 15/16). Written informed consent was obtained from the donors. Endothelial identity was confirmed by positive staining for von Willebrand factor (vWF; immunoglobulin fraction, rabbit anti-human, 0.7 μg/ml; Dako, Glostrup, Denmark) and negative staining for CD90 (clone ASO2, 0.1 μg/ml, mouse IgG_1_; Dianova, Hamburg, Germany), smooth muscle actin (clone 1A4, 0.2 μg/ml, mouse IgG_2a_, Dako) and desmin (clone D33, 0.4 μg/ml, mouse IgG_1_, Dako).

Primary human pulmonary microvascular endothelial cells (HPMEC) are microvascular endothelial cells purchased from PromoCell (Heidelberg, Germany). HPMEC were cultured in Endothelial Cell Growth Medium MV (PromoCell). Both cell types were used between passages 4 to 7 for the experiments described below.

The human monocytic cell line THP-1 was purchased from ATCC (American Type Culture Collection, Nr. TIB-202, Wesel, Germany) and was cultured in RPMI-1640 media (Sigma-Aldrich) supplemented with 10% FBS (PAA), 20 mM HEPES (Sigma-Aldrich), and 100 μM P/S (Sigma-Aldrich). Cells were maintained at densities between 0.2 and 1.0x10^6^ cells/ml in culture. All cell cultures were maintained in a humidified 5% CO_2_−95% air incubator at 37°C.

All cell types were checked for mycoplasma infection using Mycoplasma Detection Kit from Biotool (Munich, Germany).

### Preparation of conditioned medium (CdM)

THP-1 monocytes were stimulated with LPS for 4 h, and the supernatant was used for the stimulation of HUVEC and HPMEC. Briefly, THP-1 monocytes (1x10^6^ cells/ml; ATCC) were incubated with Medium M-199 for 48 h (Sigma-Aldrich) containing 10% human AB-plasma (Humanplasma, Retz, Austria), CaCl_2_ (Sigma-Aldrich), 15 IU/ml heparin (Baxter), and 100 μM P/S (Sigma-Aldrich). Additionally, 10 ng/ml LPS from *Pseudomonas aeruginosa* (Sigma-Aldrich) was added for 4 h at 37°C and 5% CO_2_. An unstimulated control was incubated in parallel with the stimulation experiments. Cell suspensions were centrifuged at 1,000 x *g* for 5 min and the supernatant was stored at -70°C until stimulation of HUVEC and HPMEC. A measured TNF-α concentration of 600 pg/ml guaranteed successful stimulation of THP-1. We chose 600 pg/ml as threshold, because in a study of serum levels of TNF-α in patients developing sepsis Damas et al. found a mean level of TNF-α of 701 ± 339 pg/ml [14]. In addition, the stimulation of THP-1 cells, PMBC, and monocytes with LPS resulted in comparable TNF-α expression patterns with a peak at 4 h and a subsequent decrease over time. Monocytes secreted higher amounts of TNF-α than PBMC and THP-1 cells (3460 ± 245 pg/ml versus 1960 ± 1365 pg/ml versus 1440 ± 696 pg/ml at 4 h, respectively) [15]. The data from both studies are the basis for the used cutoff of 600 pg TNF-α/ml [14,15].

### Stimulation of HUVEC and HPMEC

HUVEC and HPMEC were grown to confluency, washed with 5 ml M-199/HEPES/PS and stimulated with TNF-α (800 pg/ml; R&D Systems, Minneapolis, USA) or CdM. At defined time points, supernatants were centrifuged at 1,000 x *g* and stored at -70°C until analysis.

### RNA extraction

miRNA for microarray experiments and real-time PCR analysis was isolated from cells using the Exiqon #300110 miRCURY RNA Isolation kit (Exiqon, Vedbæk, Denmark) according to the manufacturer’s instructions. Quantity and quality of the RNA were determined with a Quant-iT-RNA Assay kit from Invitrogen (Carlsbad, California, USA).

### miRNA array hybridization and data analysis

Labelled RNA (2 μg) was hybridized at 56°C to a miRCURY LNA Array (Exiqon) in a hybridization chamber using a microarray hybridization solution (Exiqon). The miRCURY LNA Array contains 1,891 capture probes covering all miRNAs annotated in miRBase 14. The capture probes are Locked Nucleic Acid (LNA)-enhanced oligonucleotides that are T_m_-normalized by varying the LNA content and length.

The hybridization and analysis service from Exiqon was used for scanning and data acquisition, and analysis. miRNAs that indicated a 1- to 1.5-fold change in expression in HUVEC and HPMEC after the stimulation with CdM were considered to present a desired candidate for further experiments.

### Real-time PCR analysis

Real-time PCR analysis was performed in order to verify miRNA array results. Isolated RNA was converted to cDNA with the Ambion High Capacity cDNA kit (Applied Biosystems, Ambion, Foster City, CA, USA), and the appropriate TaqMan Gene Expression Assay (Applied Biosystems) was used to monitor the changes in expression using an iCycler Real-time PCR System (Bio-Rad, Vienna, Austria).

### Transfection with miRNA inhibitors

Inhibitors for the selected miRNAs were purchased from Qiagen (Hilden, Germany) and transfected with HiPerFect (Qiagen) according to the manufacturer’s instructions. Briefly, 3 μl of HiPerFect Transfection Reagent was added to the diluted inhibitors for miR-146a, miR-146b, and miR-155 (25 mM and 50 mM; in 100 μl M-199/ HEPES/FBS/ECGS/heparin) and mixed by vortexing for 10 s. The samples were incubated for 10 min at room temperature to enable the formation of the transfection complex. The transfection complexes were added dropwise onto HUVEC (6x10^4^ cells/well; 24-well plate), and the plate was gently swirled to ensure distribution of the transfection complexes. After 3 h 400 μl of M-199/ HEPES/FBS/ECGS/heparin was added per well. The transfected cells were stimulated with CdM for 24 h after the transfection process. Mock inhibitor miRNA served as a control.

### Assessment of cytokines and heat shock proteins

TNF-α, IL-1β, IL-6, IL-8, and IL-10 were measured by BioPlex, BioRad (Luminex Technology). Expression of HSP10, HSP27, and TXA2 was analyzed by ELISA (Bender-MedSystems, Vienna, Austria).

### Statistics

Statistical analysis was performed using SPSS IBM Statistics 21 (SPSS Inc., Chicago, IL, USA). The results are expressed as the means ± standard deviation (SD). For statistical analysis, Student’s *t*-test (unpaired, two-tailed) was performed. *P*-values are indicated as *P< 0.05 and **P<0.001, and were considered to be significant.

## Results

### Cytokine profile of CdM

**Fig 1:**
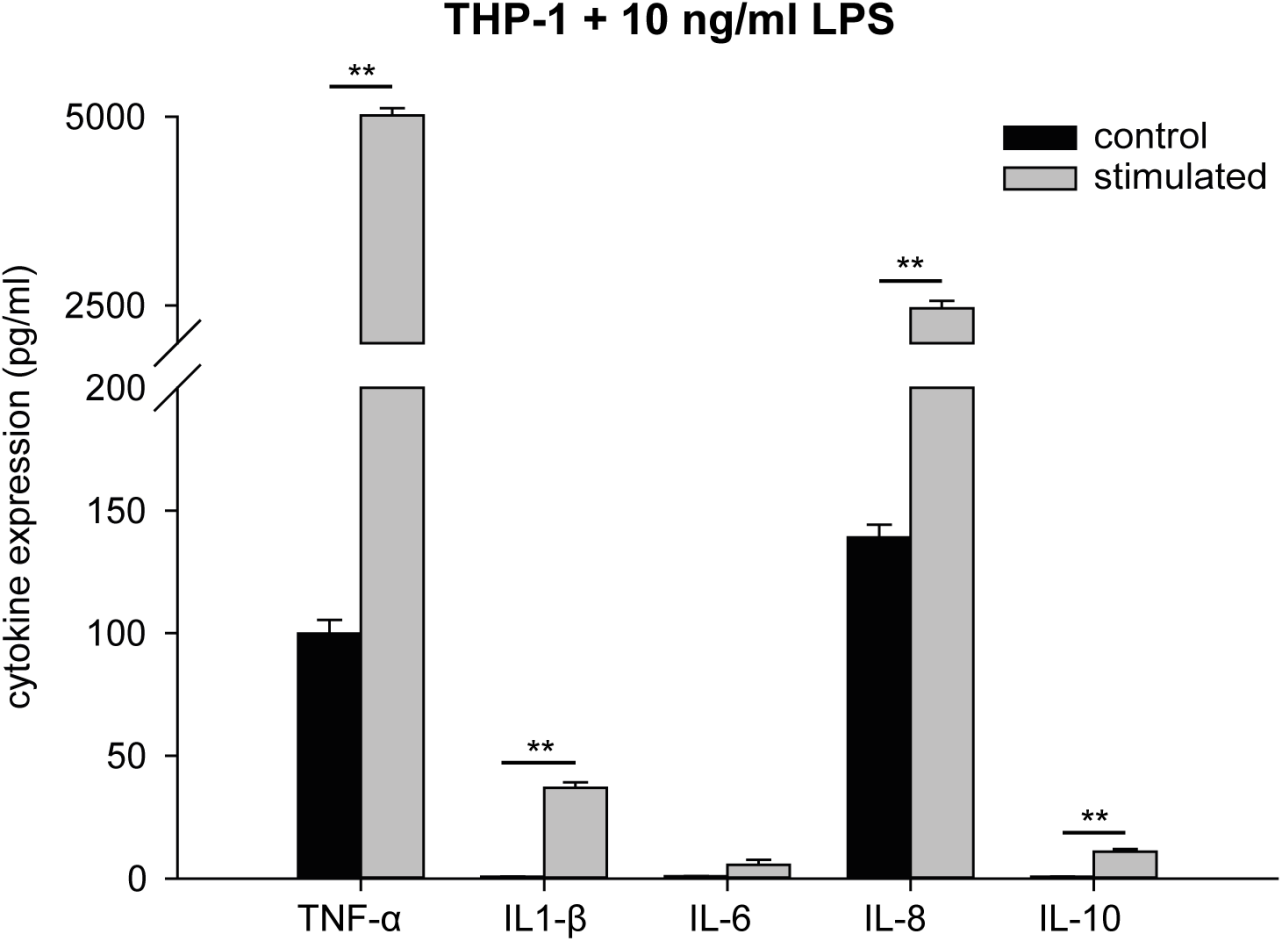
Cytokine profile of stimulated THP-1. THP-1 monocytes were stimulated with 10 ng/ml LPS, and expression of TNF-α, IL-1β, IL-6, IL-8, and IL-10 was measured after 4 h. The supernatant was collected and used as CdM for stimulation of HUVEC. The data represent the means ± SD (n = 3). **P < 0.001.

Stimulation of THP-1 monocytes with 10 ng/ml LPS for 4 h led to an increase in the expression of TNF-α (5,317 ± 98 pg/ml; control 100 ± 6 pg/ml) and IL-8 (2,464 ± 99 pg/ml; control 139 ± 12 pg/ml). Expression of the cytokines IL-1β, IL-6, and IL-10 was slightly increased (IL-1β, 36.9 ± 2.3 pg/ml compared to control 0.8 ± 0.1 pg/ml; IL-6, 5.6 ± 2.0 pg/ml compared to control 0.9 ± 0.1 pg/ml; IL-10, 11.0 ± 1.0 pg/ml compared to control 0.7 ± 0.1 pg/ml) (Fig 1, S1 Table).

### Effect of CdM on cytokine expression of HUVEC

**Fig 2:**
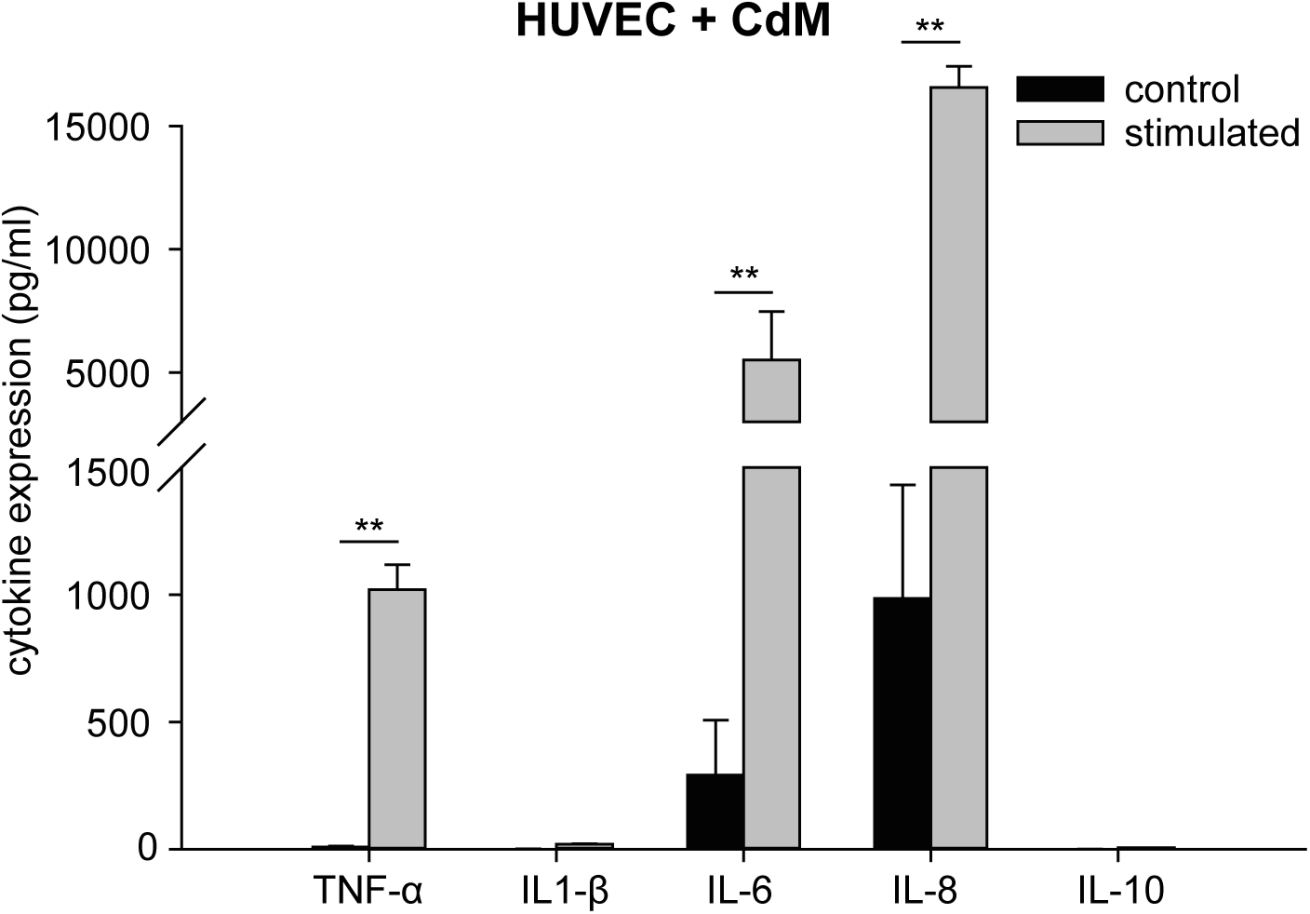
Cytokine profile of CdM-stimulated HUVEC. HUVEC were stimulated with CdM and expression of TNF-α, IL-1β, IL-6, IL-8, and IL-10 was measured after 16 h. The data represent the means ± SD (n = 3). **P < 0.001.

HUVEC showed an increase in the expression of TNF-α, IL-6, and IL-8 when cultured in CdM for 16 h compared to the control (CdM without cells) (TNF-α, 1,020 ± 98 pg/ml compared to control 10 ± 3 pg/ml; IL-6, 5,509 ± 291 pg/ml compared to control 291 ± 216 pg/ml; IL-8, 16,569 ± 855 pg/ml compared to control 985 ± 447 pg/ml). Concentrations of IL-1β (20.35 ± 1.29 pg/ml compared to control 0.74 ± 0.25 pg/ml) and IL-10 (6.28 ± 0.43 pg/ml compared to control 0.42 ± 0.23) increased slightly compared to CdM control (Fig 2, S2 Table).

### Comparison of TNF-α and CdM as stimuli for HUVEC

**Fig 3:**
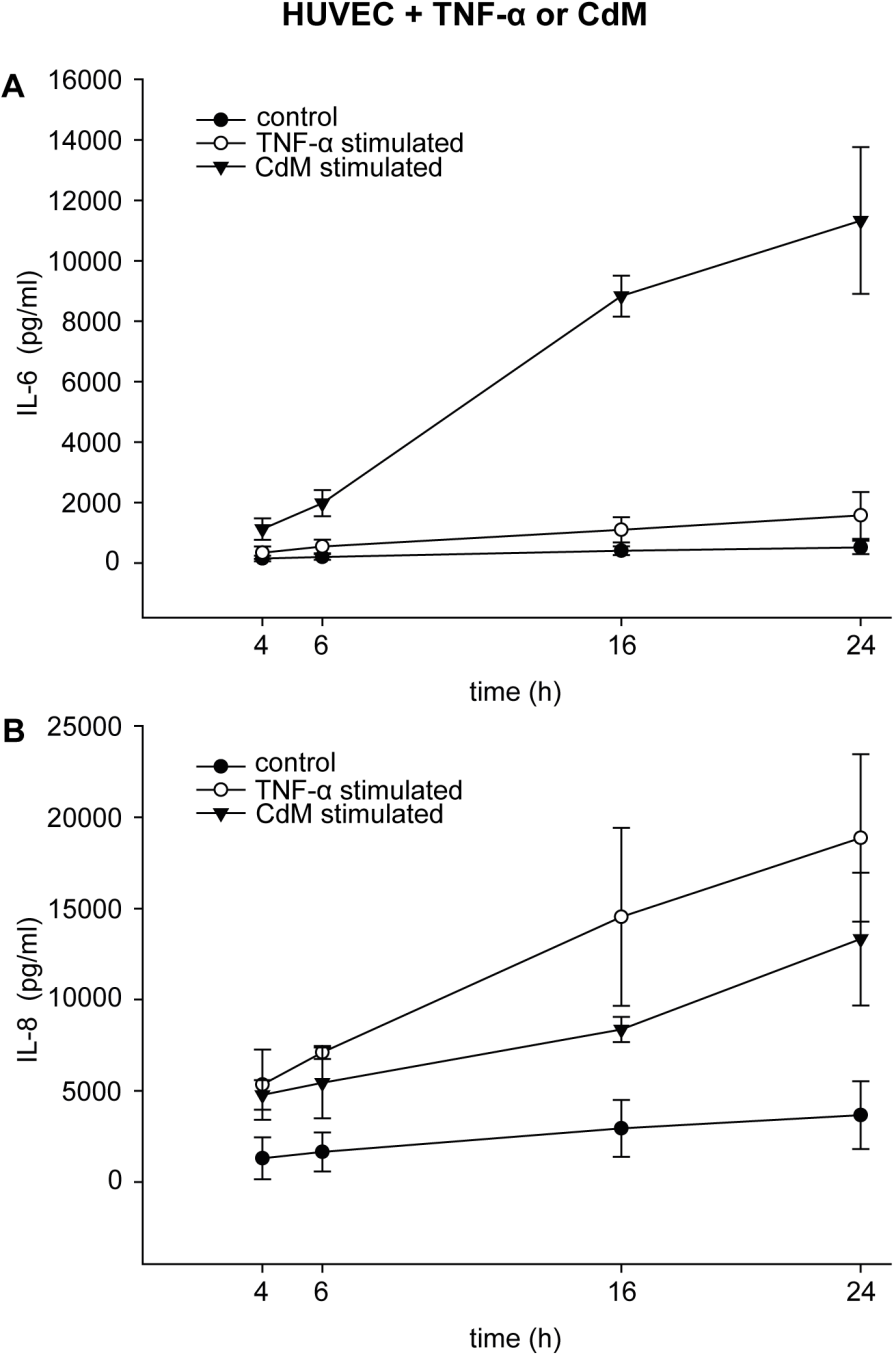
Different expression of IL-6 and IL-8 of HUVEC stimulated with CdM compared to TNF-α. HUVEC were stimulated with CdM or 800 pg TNF-α /ml, and expression of IL-6 (A) and IL-8 (B) was measured after 4 h, 6 h, 16 h, and 24 h. The data represent the means ± SD (n = 3).

Stimulation of HUVEC with TNF-α for 16 h and 24 h slightly increased the expression of IL-6 at 1,101 ± 417 pg/ml and 1,577 ± 766 pg/ml, respectively compared to the corresponding controls at 411 ± 145 pg/ml and 519 ± 221 pg/ml. In contrast, stimulation of HUVEC with CdM for 16 h led to an 8-fold increase (8,831 ± 682 pg/ml), and stimulation for 24 h led to a 7-fold increase in IL-6 (11,331 ± 2,429 pg/ml) compared to stimulation with TNF-α (Fig 3A, S3 Table). However, IL-8 expression of HUVEC stimulated with TNF-α exceeded the 1.7-fold expression of HUVEC stimulated with CdM. Stimulation of HUVEC with TNF-α for 24 h led to an expression of 18,871 ± 4,588 pg IL-8/ml compared to 13,319 ± 3,643 pg IL-8/ml when stimulated with CdM (control 3,668 ± 1,857 pg/ml) (Fig 3B, S3 Table).

### miRNA expression profile of stimulated HUVEC and HPMEC

**Fig 4:**
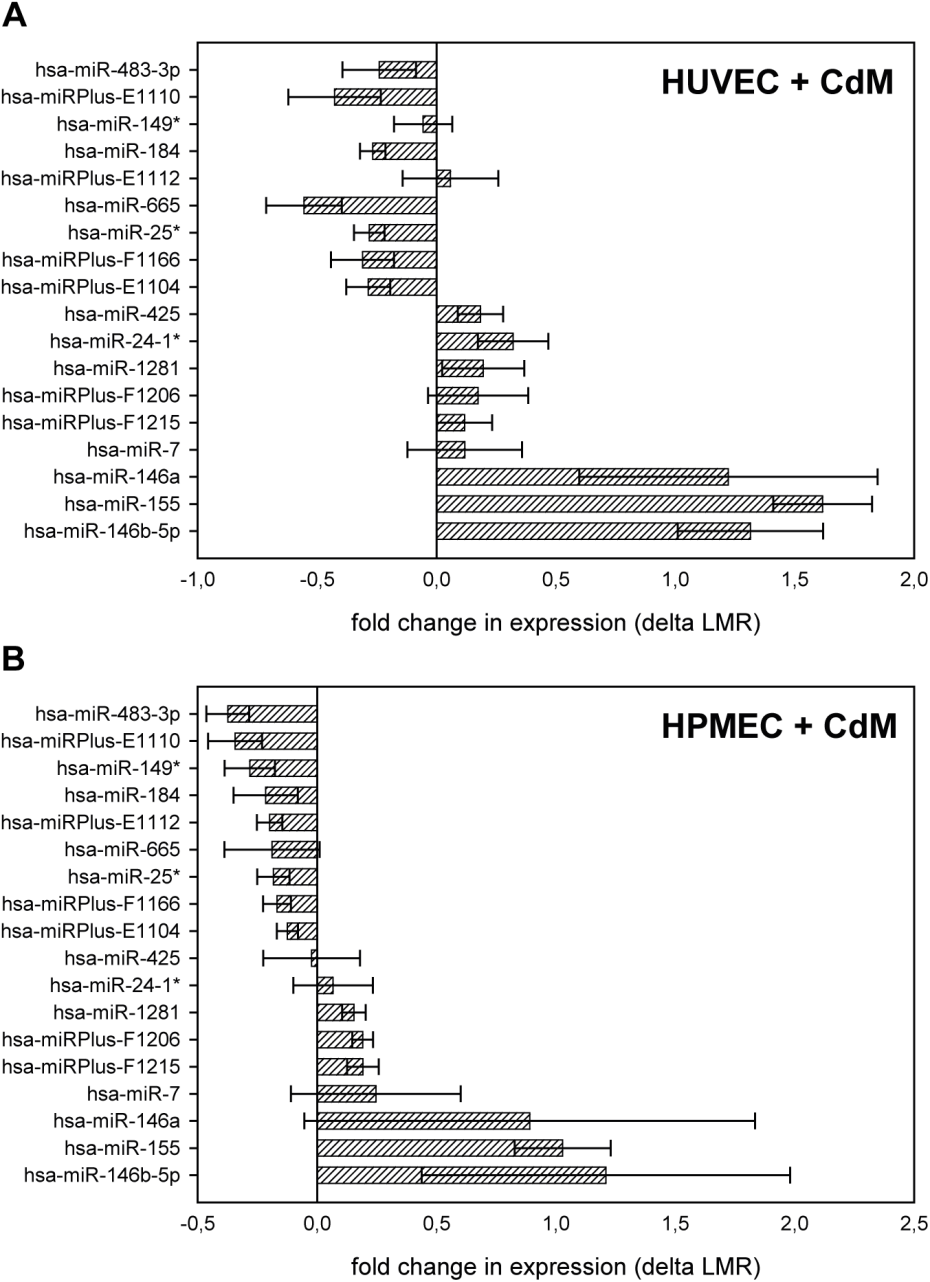
miRNA expression changes in HUVEC and HPMEC stimulated with CdM. We stimulated HUVEC (A) and HPMEC (B) with CdM for 16 h and investigated 1,891 miRNAs by array analysis. The bars indicate fold changes in miRNA expression (delta LMR) compared to the unstimulated control. The data represent the means ± SD (n = 3).

Stimulation of HUVEC and HPMEC with CdM for 16 h resulted in changes in the expression of 18 out of 1,891 measured miRNAs. Array analysis indicated an increased expression of 10 miRNAs (miRPlus-E112, miR-425, miR-24-1, miR-1281, miR-Plus-F1206, miRPlus-F1215, miR-7, miR-146a, miR-155, and miR-146b-5p) and a decreased expression of 8 miRNAs (miR-483-3p, miRPlus-E1110, miR-184, miR-665, miR-25, miRPlus-F1166, and miRPlus-E1104) in HUVEC (Fig 4A, S4 Table), whereas the expression of 8 miRNAs (miR-24-1, miR-1281, miR-Plus-F1206, miRPlus-F1215, miR-7, miR-146a, miR-155, miR-146b-5p) was increased and that of 10 miRNAs (miR-483-3p, miRPlus-E1110, miR-149, miR-184, miRPlus-E112, miR-665, miR-25, miRPlus-F1166, miRPlus-E1104, and miR-425) decreased in HPMEC after stimulation with CdM (Fig 4B, S4 Table). The expression pattern after stimulation was only slightly different between the two cell types. miR-146a, miR-146b, and miR-155 exhibited the most differences in expression pattern after treatment with CdM in both cell types, HUVEC and HPMEC. These miRNAs indicated a 1- to 1.5-fold change in expression in HUVEC and HPMEC after treatment. In addition, qPCR was performed in order to verify the miRNA array results (data not shown, S4 Table).

### Inhibition of miR-146a, miR-146b, and miR-155 reduced expression of IL-6 and IL-8 in CdM-stimulated HUVEC

**Fig 5:**
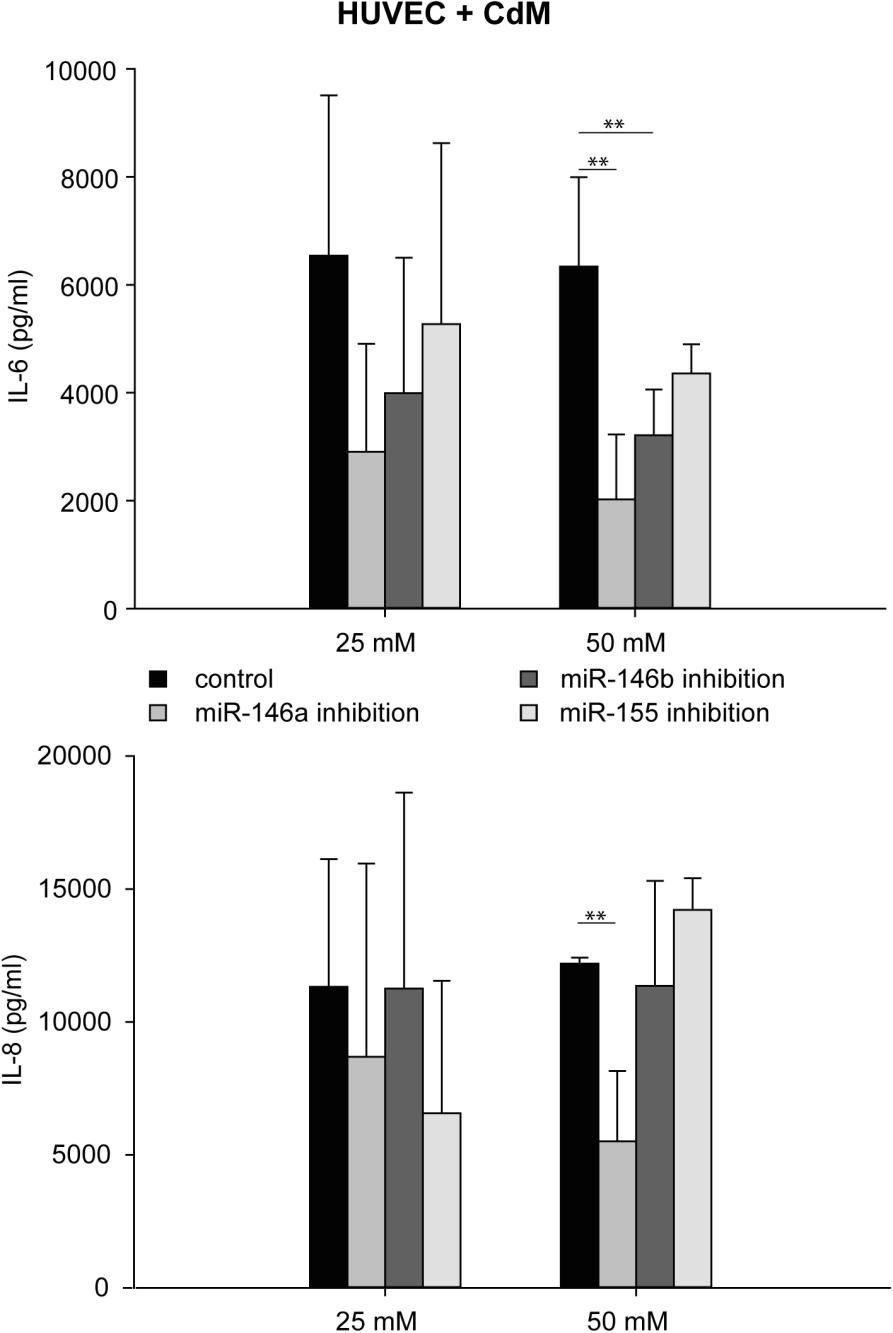
Changes in the expression of IL-6 and IL-8 in CdM-stimulated and miR-146a-, miR-146b- or miR-155-inhibited HUVEC. We stimulated HUVEC with CdM after inhibition of miR-146a, miR-146b or miR-155 and measured the expression of IL-6 and IL-8 after 16 h. We added inhibitors for miR-146a, miR-146b or miR-155 at concentrations of 25 mM or 50 mM. A mock control miRNA served as the control. The data represent the means ± SD (n = 3). **P < 0.001.

Inhibition of miR-146a, miR-146b, and miR-155 with inhibitors of miR-146a, miR-146b, and miR-155 (50 mM) reduced IL-6 and IL-8 expression of CdM-stimulated HUVEC (16 h) compared to that of the control (stimulation with CdM containing mock control miRNA). Inhibition of miR-146a led to a 68% reduced expression of IL-6 (2,021 ± 1,204 pg/ml) compared to the control (6,336 ± 1,657 pg/ml), inhibition of miR-146b resulted in a 49% reduction in IL-6 expression (3,211 ± 846 pg/ml), and inhibition of miR-155 led to a 31% reduction in IL-6 expression (4,355 ± 543 pg/ml). In contrast, inhibitor concentrations at 25 mM did not significantly reduce the concentration of IL-6 (Fig 5A, S5 Table).

In addition, inhibition of miR-146a with inhibitors for miR-146a at a concentration of 50 mM reduced the expression of IL-8 (55%; 5,506 ± 2,649 pg/ml) compared to control (12,195 ± 228 pg/ml). In contrast, inhibition of miR-155 with inhibitors for miR-155 (50 mM) increased the expression of IL-8 by 14% (14,214 ± 1,195 pg/ml). Neither inhibition of miR-146b (11,359 ± 3,945 pg/ml) with inhibitors for miR-146b with concentrations of 50 mM nor inhibition of miR-146a, miR-146b, and miR-155 with the respective inhibitors for at a concentrations of 25 mM had any significant effect on the expression of IL-8 (Fig 5B, S5 Table).

### Inhibition of miR-146a, miR-146b, and miR-155 reduced the expression of HSP10 in stimulated HUVEC but did not affect HSP27 and TXA2

**Fig 6:**
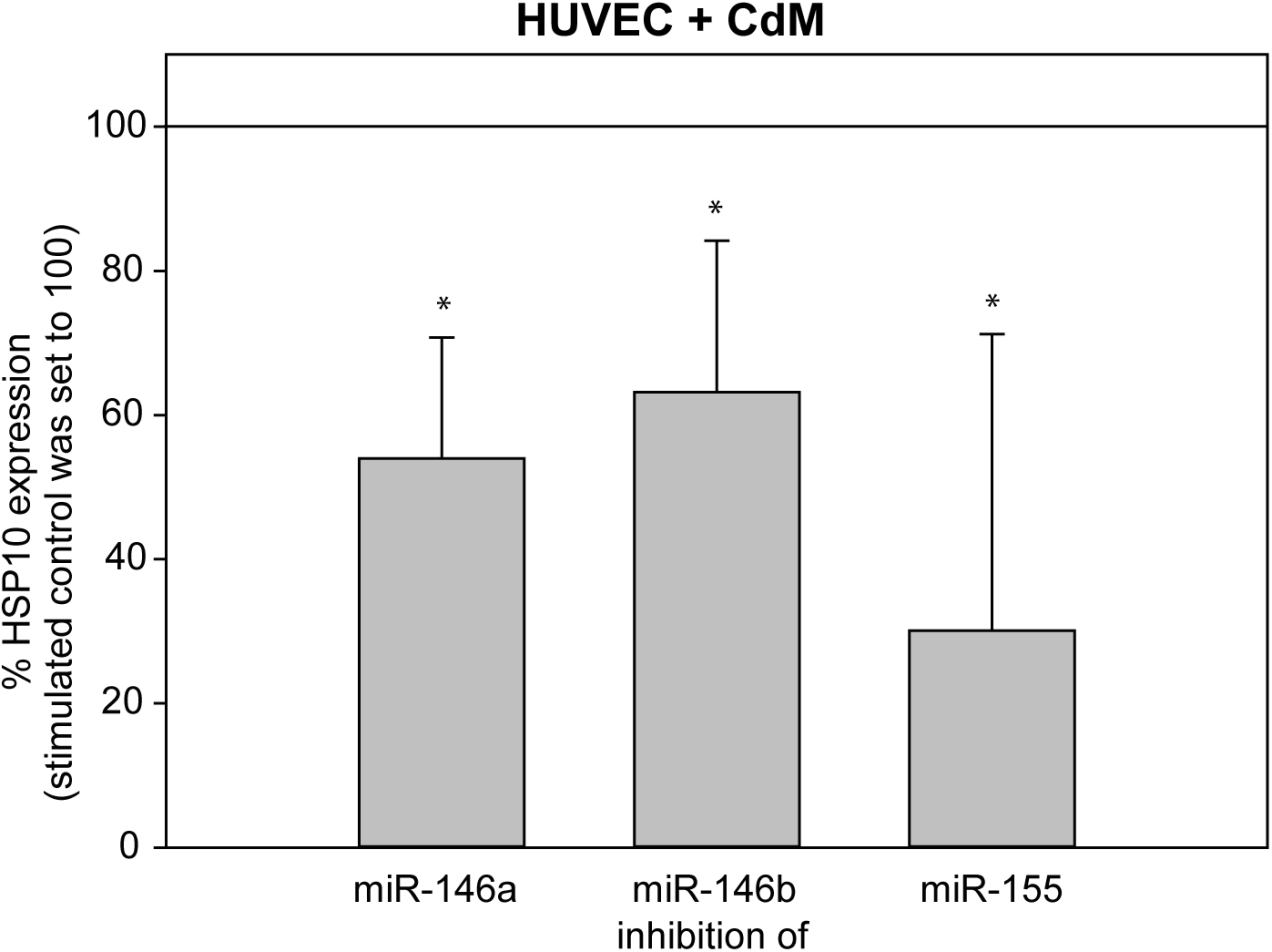
Inhibition of miR-146a, miR-146b, and miR-155 reduced the expression of HSP10 in CdM-stimulated HUVEC. We stimulated HUVEC with CdM after inhibition of miR-146a, miR-146b, or miR-155 and measured the expression of HSP10 after 16 h. CdM-stimulated HUVEC acted as a control, and their expression levels were set to 100%. The data represent the means ± SD (n = 3). *P < 0.05.

Inhibition of miR-146a, miR-146b, and miR-155 resulted in a reduced expression of HSP10 in stimulated HUVEC (Fig 6, S6 Table). miR-146a inhibition caused a 50% reduction in HSP10 release in stimulated HUVEC (8,937 ± 3,705 ng/ml) compared to the unstimulated control (17,790 ± 9,807 ng/ml), inhibition of miR-146b resulted in a 35% reduction in HSP10 expression (11,522 ng/ml ± 8,328 ng/ml), and inhibition of miR-155 led to a decrease in HSP10 concentration by 69% (5,435 ± 7,686 ng/ml).

Inhibition of miR-146a, miR-146b, and miR-155 in stimulated HUVEC did not influence the expression of HSP27 and TXA2 (data not shown, S6 Table).

## Discussion

This study aimed to identify miRNAs that are involved in the innate immune response and to assess their potential functions in the activation of the endothelium. We have shown that expression of IL-6, IL-8, and HSP10 after stimulation of HUVEC with CdM is affected by miR-146a, miR-146b, and miR-155. CdM was demonstrated as a potent stimulator for HUVEC and HPMEC, and it significantly increased the expression of TNF-α, IL-6, and IL-8. TNF-α, widely used as a stimulator for endothelial cells [16,17], was not able to increase the expression of IL-6 compared to the control after 16 h and 24 h of stimulation. It has already been reported in the literature that TNF-α only in synergy with LPS leads to an effective activation of NF-κB and its translocation into the nucleus, which results in an increased expression of endothelial cell adhesion molecules [18]. Blocking TNF-α and IL-1β in the plasma from LPS-treated human whole blood was shown to reduce the increase in endothelial permeability in a stimulation model of endothelial cells [19]. These data are in accordance with our findings and lead us to assume that multifactorial stimulation is necessary for the sufficient activation of endothelial cells.

miR-146a, miR-146b, and miR-155 displayed an almost 2-fold increase in expression after stimulation of HUVEC and HPMEC with CdM compared to the control. miR-146 transcription is activated by LPS, TNF-α, and IL-1β via NF-κB and targets TNF receptor-associated family (TRAF) 6 and IL-1 receptor-associated kinase (IRAK) 1, implicating it as a negative regulator in the activation of monocytes and macrophages [9,20,21]. It has also been reported that miR-146 and miR-155 are upregulated in Raw 264.7 macrophages in response to LPS to target transcription for several proteins involved in LPS signaling and increase the rate of translation of TNF-α transcripts [22,23]. miR-155, similar to miR-146, is involved in the negative regulation of immune responses to LPS. miR-155 not only represses but also favors the activation of the LPS/TNF-α pathway by enhancing translation of TNF-α in macrophages [22]. However, the function of miRNAs in the activation of the endothelium during inflammation remains rather elusive. miR-31 and miR-17-3p are known to be induced by TNF-α in endothelial cells and specifically target both E-selectin and ICAM-1 by feedback regulation in inflammation [12]. miR-181b serves as a potent regulator of proteins involved in NF-κB signaling in the vascular endothelium. In addition, reduced levels of circulating miR-181b have been found in patients with sepsis [24]. Although the role of miR-146 and miR-155 has been intensively investigated in monocytes and macrophages, little is known about the expression of miR-146 and miR-155 in the endothelium. Profiling of 843 human miRNAs revealed that miR-146 and miR-155 are not expressed by endothelial cells [25]; in endothelial cells, miR-146 is associated with cell senescence, and it enhances angiogenic activity, acts as a negative regulator of inflammation and represses the pro-inflammatory NF-κB pathway as well as the MAP kinase pathway [26–28]. Controversially, our data lack signs of negative feedback regulation of inflammation. We found elevated expression of miR-146 after stimulation of endothelial cells and a profound effect on the expression of pro-inflammatory IL-6 and IL-8. Cheng et al. observed a negative feedback regulation by miR-146 after stimulation of endothelial cells with IL-1β [26]. As already mentioned, our data indicate the impact of the stimulus on the study outcomes. Stimulation with single factors compared to multi-factorial stimulation could be an explanation for the contradictory results. However, miR-155 mediates endothelial inflammation and decreases the expression of NFκB subunit p65 and adhesion protein in TNF-α-stimulated endothelial cells; miR-155 has also been identified to be flow sensitive *in vitro* and is therefore designated, among others, as mechano-miR [29,30]. In addition, microparticle encapsuled miR-155 was found to be released from human aortic endothelial cells after TNF-α treatment [31]. These studies are in concordance with our results indicating that miR-155 is involved in the enhanced expression of the pro-inflammatory cytokines IL-6 and IL-8.

HSPs have important effects on homeostasis and cell survival, and they activate innate and adaptive immune responses. HSP10 has been reported to have tumor growth promoting properties but plays an important role in the inhibition of immunoinflammatory reactions [4,32,33]. The *in vitro* administration of recombinant HSP10 seems to inhibit LPS-induced NF-κB activation, leading to decreased concentrations of inflammatory cytokines and chemokines [5]. We have observed an increase in HSP10 expression in HUVEC after stimulation with CdM. Inhibition of miR-146 and miR-155 significantly reduced the expression of HSP10 and enables us to conclude a positive regulation of HSP10 by miR-146 and miR-155. Therefore, miR-146 and miR-155 strengthen the notion of inhibition of inflammation by HSP10.

*In vitro* investigations of inflammation and sepsis relevant pathological mechanisms pose a challenge to the experimental design. Although our studies were performed with primary endothelial cells cultured in medium with human plasma the experiments do not reflect the multi-factorial situation of a whole body inflammation. We tried to overcome this problem partially by the set-up of an *in-vitro* sepsis model and stimulated THP-1 monocytes with LPS for 4 h to use this supernatant with all known and unknown factors for the stimulation of endothelial cells [34,35].

Our study confirmed the hypothesis that inhibition of selected miRNAs leads to an altered cytokine performance and influences the expression of HSPs in sepsis-relevant experiments. We have shown that miR-146a, miR-146b, and miR-155 are potential targets for novel therapeutic approaches in the treatment of inflammatory diseases.

## Supporting information

S1 Table Data of Fig 1. Expression of TNF-α, IL-1β, IL-6, IL-8, and IL-10 of stimulated THP-1.
(XLSX)

S2 Table Data of Fig 2. Expression of TNF-α, IL-1β, IL-6, IL-8, and IL-10 of CdM-stimulated HUVEC.
(XLSX)

S3 Table Data of Fig 3. Expression of IL-6 and IL-8 of HUVEC stimulated with CdM compared to TNF-α.
(XLS)

S4 Table Data of Fig 4. miRNA expression in HUVEC and HPMEC stimulated with CdM.
(XLS)

S5 Table Data of Fig 5. Expression of IL-6 and IL-8 in CdM-stimulated and miR-146a-, miR-146b- or miR-155-inhibited HUVEC.
(XLS)

S6 Table Data of Fig 6. Expression of HSP10 in CdM-stimulated and miR-146a-, miR-146b- or miR-155-inhibited HUVEC.
(XLSX)
